# Prof. Roux on Luxation of Elbow

**Published:** 1847-12

**Authors:** 


*Prof. Roux on Luxation of Elbow.*—There is an example of this luxation in our wards, said Professor Roux a short time since, which affords me an opportunity of offering you some remarks upon the nature of this lesion. The nature of the case was not recognized by the physician who was first called in. You have never known me, he remarked, especially at the bedside, accuse a professional brother of having committed an error; the blame, always out of place, will often be unmerited when it concerns a traumatic affection of the elbow. The articulation is studded with osseous projections which cannot be felt, or which may be confounded the one with another, because of swelling, particularly in women, and still more among children, when they are but slightly disclosed. Our best surgical treatises are, moreover, as regards the diagnosis of these luxations, indefinite and unsatisfactory. For example, they tell you that in the displacements backwards the forearm is always flexed, and that this flexion, with the projection of the olecranon behind, will constitute the two essential characters of the lesion. Now, very often the position of the limb is nearer that of extension than flexion, and in trusting to the pretended constancy of this sign you will be led into error of diagnosis. The displacement is overlooked, especially in children and women, where the osseous projections, as we have said, and the muscles are less clearly defined beneath a tumefaction which, although varying in degree in different cases, is always present. It requires sometimes long practice to avoid this mistake. I have seen ten or twelve dislocations of the elbow thus overlooked, or, to speak more plainly, misunderstood, and left for a long time without any attempts being made to reduce them. It is a fortunate circumstance that after many weeks, and even after many months, we may still succeed in reducing them. If we fail to do so, the motion of the joint is in a great degree lost, and the limb remains permanently extended just as if it were composed of one piece.

I will cite you a case of success: I choose it among those that I have myself observed, because, apart from the pathological interest which attaches to it, there is something of romance connected with it. It was twelve years since, in a foreign country. I was at Milan, having just returned from Pavia, (where, I may remark in passing, I saw the head of Scarpa macerating, unhonored, among other anatomical specimens in a tub.) On my return from this excursion I found some gendarmes awaiting me at my hotel. At that time recent troubles prompted stringent measures of precaution concerning travellers. For a moment I was a little alarmed. The commandant of the detachment advanced towards me, announcing as he approached that he came to consult me about his son. As you may imagine, I was instantly relieved. His son, who was in the Austrian cavalry, had about five months before fallen from his horse and dislocated his elbow. The nature of the lesion escaped the first physicians who were called in. Panezza, and the successor of Scarpa, to whom the patient afterwards presented himself, said they had seen the luxation too late, and made no attempt to reduce it. I examined the elbow; the displacement was evident; there was scarcely the least mobility between the bones. I told the father of the patient that I had often reduced ancient luxations, but none had been in a state similar to this; that I was desirous of attempting it, however, without warranting success; that at any rate there was no danger from prudently directed efforts. I employed a number of robust men in extension and counter-extension, and succeeded. I have seen since many similar examples of success.

Carried too far, these attempts at reduction may produce serious accidents. Unnatural adhesions may be formed, and I only wonder that we do not more frequently fail. I myself have never observed any other accidents here than the fracture of the olecranon; it occurred at the moment when the flexion of the forearm completed the reduction. There was no laceration of the integuments, nor any other unpleasant consequences; but the joint regained its mobility only very incompletely; extension remained limited, which, however, offered less inconvenience than if the limit of motion had been in the opposite direction.

Fractures of the elbow also present great difficulty of diagnosis. It is often hard to pronounce upon the seat, direction, and sometimes even upon the existence of a solution of continuity of the bone. This is easily understood: the least derangement in a machine—the breaking of one of its wheels, the bending of one of its teeth—and its movements are clogged; the apparatus stops. The same is the case with the articulations, and especially with those that are unequal, as of the elbow. We feel that it is not always an easy matter to perceive the spoke of the wheel which has suffered, and the exact alteration which it has experienced. We must be careful in the diagnosis and reserved in the prognosis, remembering that simple contusions of the elbow, especially among children, may lead to anchylosis. If in practice credit has sometimes been given us for cures in which nature has largely contributed to success; more often still have reverses been laid at our door for which the gravity of the lesion alone was responsible. One of my best pupils and one of our most distinguished provincial surgeons, M. Fleury, Jr., had given the most assiduous and enlightened care to a fracture of the elbow; pronation and extension unfortunately remained embarrassed, and it was impossible for me to persuade the patient that it was not the fault of the surgeon. You must not be too sensitive, but prepare yourselves for similar reproaches which the most able have received before you.—*Ibid.*

